# A COX-2-Targeted Platinum(lV) Prodrug Induces Apoptosis and Reduces Inflammation in Bladder Cancer Models

**DOI:** 10.3390/ph18081185

**Published:** 2025-08-12

**Authors:** Ya Li, Siyang Liu, Meng Zhou, Zihan Zhao, Dongfan Song, Hongqian Guo, Rong Yang

**Affiliations:** 1 Department of Urology, Nanjing Drum Tower Hospital, Clinical College of Nanjing University of Chinese Medicine, Nanjing 210008, China; 13545350158@163.com (Y.L.); mengzhou3@126.com (M.Z.); 2 Department of Urology, Nanjing Drum Tower Hospital, Affiliated Hospital of Medical School, Nanjing University, Nanjing 210008, China; 161230016@smail.nju.edu.cn (S.L.); doctorzzh@smail.nju.edu.cn (Z.Z.); 3 State Key Laboratory of Coordination Chemistry, Chemistry and Biomedicine Innovation Center (ChemBIC), School of Chemistry and Chemical Engineering, Nanjing University, Nanjing 210023, China; sdf_ms@nju.edu.cn

**Keywords:** bladder cancer, Pt(IV), naproxen(NPX), DNP, cyclooxygenase-2 (COX-2)

## Abstract

**Background**: Bladder cancer is a common and heterogeneous malignancy of the urinary tract. Traditional chemotherapy using bivalent platinum drugs such as cisplatin(CDDP) is often limited by severe side effects and acquired resistance. To overcome these limitations, we explored a novel Pt(IV) prodrug, DNP, designed to release both cytotoxic cisplatin and the anti-inflammatory cyclooxygenase-2 (COX-2) inhibitor naproxen(NPX). **Methods**: We evaluated the cytotoxic activity of DNP using both two-dimensional (2D) monolayer and three-dimensional (3D) spheroid models of bladder cancer cells. Transcriptomic analysis via RNA-seq identified apoptosis- and inflammation-related signaling pathways modulated by DNP. RNA-seq-based transcriptomic profiling revealed that DNP regulates signaling pathways associated with apoptosis and inflammation. The anti-inflammatory effects were evaluated using a lipopolysaccharide (LPS)-induced macrophage model, while the in vivo antitumor efficacy was assessed in an orthotopic MB49 bladder cancer model. **Results**: Compared with CDDP, DNP significantly increased intracellular platinum accumulation and exhibited superior cytotoxicity. It effectively inhibited tumor proliferation, induced apoptosis, and attenuated inflammation both in vitro and in vivo. **Conclusions**: These findings suggest that DNP exerts dual antitumor effects through enhanced delivery of cytotoxic and anti-inflammatory agents, offering a promising strategy for bladder cancer therapy.

## 1. Introduction

Bladder cancer ranks among the most prevalent malignancies of the urinary tract, with significant clinical and economic burdens globally [1]. Preliminary studies indicate that non-muscle-invasive bladder cancer (NMIBC) accounts for 70–75% of cases, muscle-invasive bladder cancer (MIBC) comprises 20–25%, while metastatic disease represents approximately 5% [2,3,4]. Despite the high treatment costs, clinical outcomes for bladder cancer remain suboptimal. Within 5 years, 31–78% of NMIBC patients experience recurrence, while 10–15% progress to MIBC, resulting in a 36–48% reduction in 5-year survival rates [5]. Cisplatin (CDDP)-based chemotherapy remains the first-line treatment for muscle-invasive bladder cancer (MIBC) patients. However, due to the high tumor heterogeneity characteristic of bladder cancer, approximately 40% of patients fail to derive clinical benefit from conventional therapy and even experience persistent disease progression [6]. Based on the side effects associated with chemotherapy (e.g., high nephrotoxicity), patients who tolerate this treatment regimen usually receive only short-term treatment. Therefore, the development of new platinum-based chemotherapeutic agents with fewer toxicities but better therapeutic efficacy deserves our more attention, and Pt(IV) prodrugs have come onto the stage. The Pt(IV) prodrug design paradigm promises to address this clinical challenge through rational axial ligand modification, enabling simultaneous delivery of multimodal payloads while preserving the DNA-damaging platinum core.

Cyclooxygenase-2 (COX-2) is an inducible enzyme correlated with inflammatory diseases and cancer [7]. Normally, the expression of COX-2 in tissues is low, while in inflammation, tumor and other conditions, the expression of COX-2 is significantly elevated [8]. Studies have shown that high COX-2 expression in bladder cancer correlates with higher stage grading of the tumor and worse prognosis of advanced bladder cancer [9,10,11]. COX-2 also modulates arachidonic acid metabolites to help cancer cells resist apoptosis, cause inflammation, and grow new blood vessels [12,13]. Thus, COX inhibitors, as known as nonsteroidal anti-inflammatory drugs (NSAIDs) may reduce inflammation by inhibiting COX-2 expression, thereby reducing tumor growth and lowering cancer risk and mortality [14,15,16,17]. The pervious study had shown that the Pt(IV) prodrug DNP, using the NSAID naproxen (NPX) as an axial ligand, has high cytotoxicity and anti-inflammatory properties in the treatment of breast cancer [18]. Given the pronounced inflammatory infiltration characteristic of bladder cancer pathogenesis, we performed a preliminary investigation into the anti-tumor activity of the Pt(IV), DNP, marking the first application of this prodrug in bladder cancer models.

## 2. Results

### 2.1. Synthesis and Characterization of DNP

**Figure 1:**
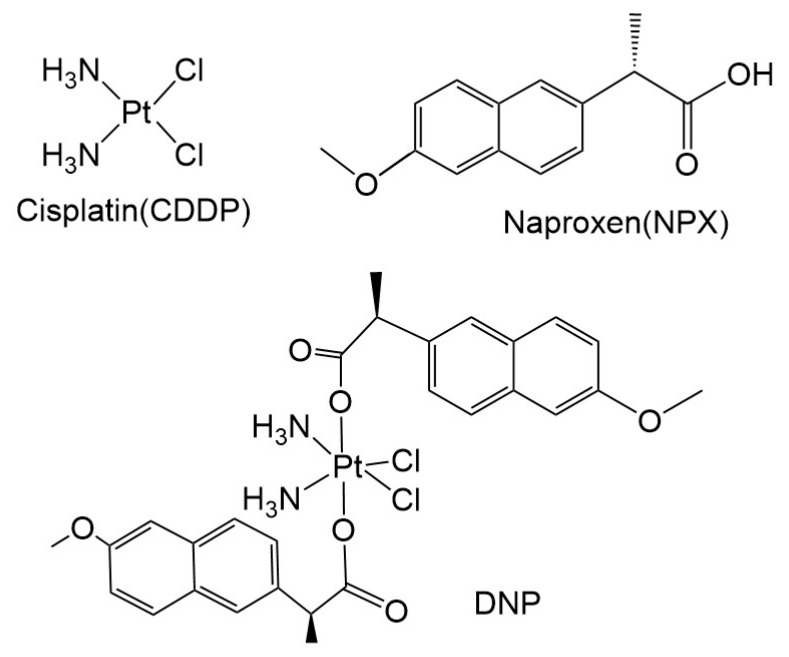
Structures of DNP, NPX, and CDDP Compounds.

The DNP was synthesized as previously reported [18] (Figure 1). Briefly, the cisplatin and naproxen were utilized as starting materials to synthesize DNP through a direct reaction between oxoplatin and NPX in dimethylsulfoxide (DMSO) solution, with the detailed synthetic route illustrated in Scheme S1. Structural confirmation was analyzed through multinuclear NMR (^1^H, ^13^C, ^195^Pt, Figures S1–S3) and high-resolution ESI-MS (Figure S4). The purity of the compound reached 98.5% (Figure S5), which can be used for subsequent experiments.

### 2.2. Cytotoxicity of DNP In Vitro

**Figure 2:**
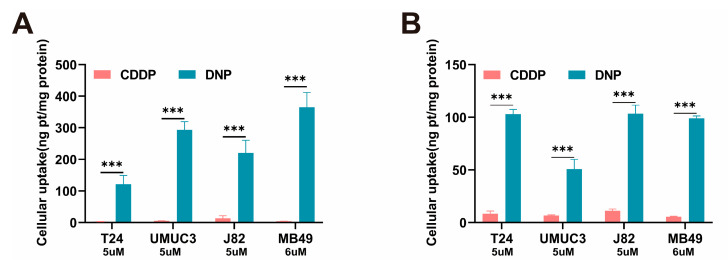
Cellular uptake of Pt (**A**) and quantification of DNA-bound Pt (**B**) in bladder tumor cells after exposure to CDDP and DNP (5μM) for 12 h (*n* = 3). *** *p* < 0.001.

Firstly, based on the CCK8 assay, we evaluated the cytotoxicity of DNP and CDDP with three human bladder tumor cell lines (T24, UMUC3, J82) and one murine-derived cell line (MB49). Half-maximal inhibitory concentrations (IC_50_) of several drugs indicated that DNP has greater cytotoxicity comparing to cisplatin and its ligand NPX (Table 1). In addition, the free NPX ligand showed little cytotoxicity and was, therefore, excluded from further studies. The accumulation of platinum drugs in tumor cells is a key indicator of their anti-tumor ability [19]. Thus, we further investigated the cellular uptake of Pt and the platinization of nuclear DNA by inductively coupled plasma mass spectrometer(ICP-MS). The results showed that DNP was significantly superior to CDDP, both in terms of intracellular platinum accumulation (Figure 2A, all *p* < 0.001) and DNA platinization in the nucleus (Figure 2B, all *p* < 0.001), which is also consistent with the results that DNP is more cytotoxic. It was observed that the more than 80% of the platinum ingested by T24 was directed towards the nucleus, indicating a notably elevated rate of nuclear utilization. Based on the IC_50_ values and the cellular uptake assay, we chose T24 cells, which are the most sensitive to DNP, for the subsequent experiments.

### 2.3. DNP Inhibited the Proliferation of Cells

**Figure 3:**
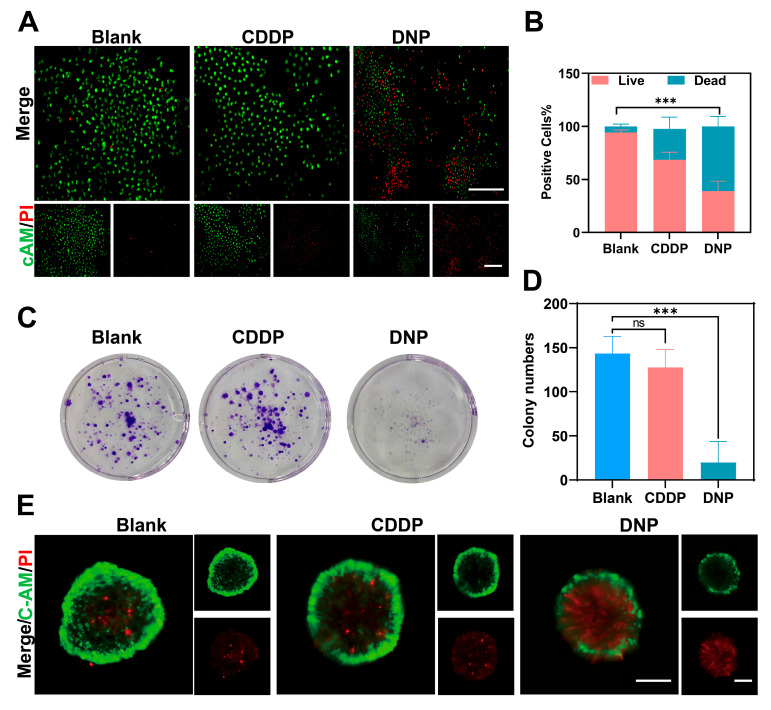
In vitro anti-tumor activity of DNP. (**A**,**B**) Live/dead cell staining assay of T24 cells treated with CDDP and DNP for 24 h (0.2 µM) (*n* = 3). Scale bar s = 100 µm. (**C**,**D**) Cell Colony formation assay and quantitative results of T24 cells treated with CDDP and DNP (0.2 µM) for 24 h (*n* = 3). (**E**) 3D cell spheroids staining after treated with cisplatin (CDDP, 5 µM) and DNP (5 µM) for 120 h (*n* = 3). Scale bars = 100 µm. ns *p* > 0.05; *** *p* < 0.001.

To further evaluate the cytotoxicity of DNP on T24 cells, we performed a live/dead cell staining assay using dyes calcein and propidium iodide (PI). The results showed that PI fluorescence intensity representing dead cells increased significantly in DNP treated T24. (Figure 3A,B, *p* < 0.01). We evaluated the proliferation of T24 cells by colony formation assay, and DNP significantly inhibited T24 colony formation compared to CDDP under the same treatment conditions (Figure 3C,D, *p* < 0.01). Considering that a three-dimensional malignant tumor cell model would more accurately reflect the response of solid tumors to treatment, we cultured T24-3D tumor cell spheres to mimic the microenvironment of solid tumors and to assess the antiproliferative activity. DNP treatment induced significant morphological alterations in T24 3D spheroids, manifesting as reduced spheroid volume and compromised structural integrity compared to cisplatin-treated counterparts. Enhanced PI fluorescence penetration demonstrated improved drug diffusion into the spheroid core, correlating with potent growth inhibition (Figure 3E). These findings collectively validated DNP’s superior cytotoxic efficacy in complex tumor models.

### 2.4. Bulk-Sequencing Analysis

**Figure 4:**
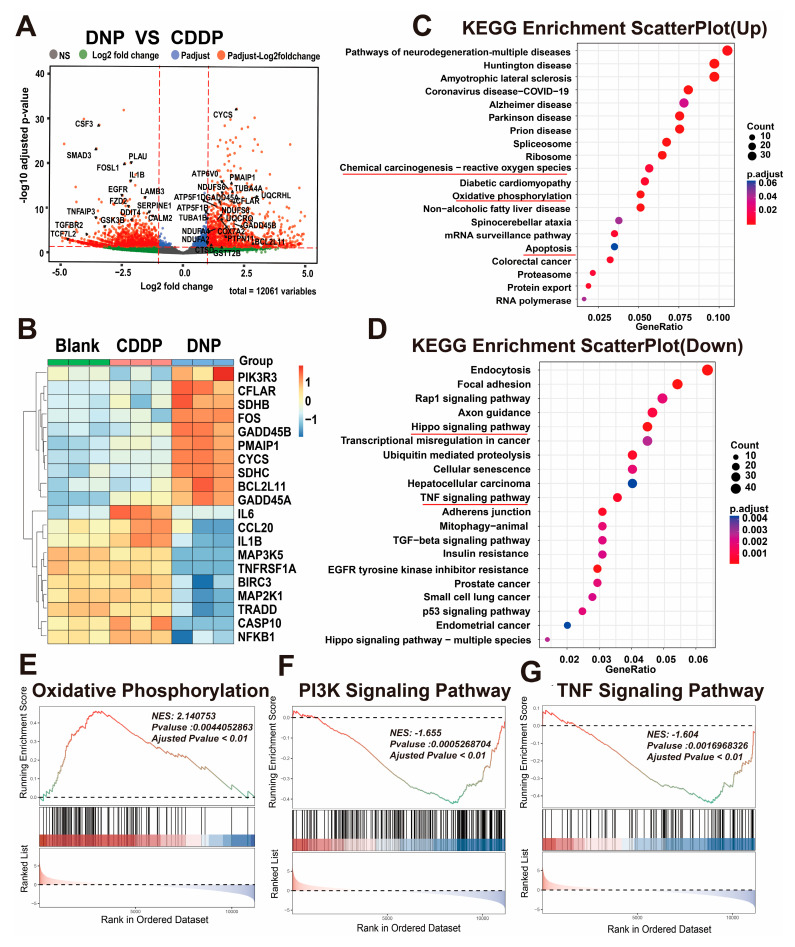
RNA-seq analysis results of the T24 treated with (DMSO) (CTRL), CDDP (2 μM) and DNP (2 μM) for 24 h. (**A**) The volcano plot displays the changes in gene expression between the DNP group and CDDP groups. (**B**) Heatmap of the 20 most significant genes in related pathways. Following a 24 h incubation period with the same concentration of CDDP and DNP (2 µM), KEGG enrichment analysis of differentially expressed genes in T24 cells was carried out (**C**,**D**). GSEA of platinum drugs after 24 h of treatment with T24 (2 µM) for genes related to reactive oxygen species (**E**), apoptosis (**F**), and inflammation (**G**).

To explore the potential mechanism by which DNP inhibits the proliferation of bladder cancer cells, we performed RNA transcriptome sequencing (RNA-seq) on T24 treated with CDDP or DNP. Compared with CDDP, there were 2540 differentially expressed genes (DEGs) tested in DNP group (|log2FoldChange| > 1, *p* < 0.05). As shown in the volcano plot, 1366 genes were down-regulated, and 1,174 genes were up-regulated (Figure 4A). Enrichment analysis of KEGG and gene set enrichment analysis (GSEA) showed that genes with significantly higher expression in DNP group were mainly enriched in the reactive oxygen species (ROS), oxidative phosphorylation and apoptosis pathways (Figure 4C,E). Previous studies have demonstrated that elevated levels of ROS lead to apoptosis of cells [20,21], which is a common anti-tumor mechanism in classical chemotherapeutic agents [22]. On the other hand, transcriptomic analysis revealed significant downregulation of TNF signaling pathway-associated genes in DNP-treated groups (Figure 4D,F,G), likely attributable to NPX-mediated suppression of inflammatory cascades through its axial coordination in the Pt(IV) prodrug architecture. Next, we performed clustering analysis on the 20 most significant DEGs, and they were mainly related to apoptosis and inflammation (Figure 4B).

### 2.5. DNP Induced Cell Apoptosis and Promoted the Generation of Reactive Oxygen Species

**Figure 5:**
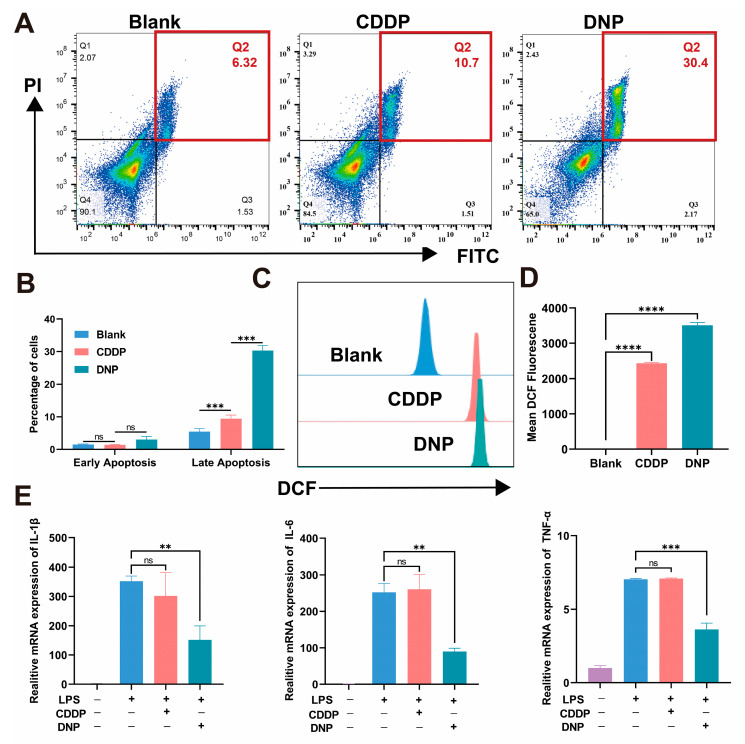
The pro-apoptotic and anti-inflammatory effects of DNP. (**A**,**B**) Induction of T24 cell apoptosis by 0.1 µM CDDP or DNP, followed by quantitative statistical analysis (*n* = 3). (**C**,**D**) The level of intracellular ROS levels in T24 cells treated with 0.2 µM CDDP or DNP for 24 h. (**E**) The expression of mRNA of TNF-α, IL-6, and IL-1β in LPS-induced macrophage activation model (*n* = 3). ns *p* > 0.05; ** *p* < 0.01; *** *p* < 0.001; **** *p* < 0.0001.

Based on the sequencing results, the pro-apoptotic and anti-inflammatory effects of DNP were further investigated. DNP-induced cell death was analyzed using flow cytometry. After a 48 h treatment, T24 were stained with fluorescein isothiocyanate (FITC) and propidium iodide to evaluate apoptosis using flow cytometry (Figure 5A,B). Consistent with the reported results, CDDP could induce apoptosis in cancer cells. Our results showed that the pro-apoptotic ability of DNP was much stronger than that of CDDP (30.4% vs. 10.7%, *p* < 0.001). Moreover, we evaluated the ROS level of T24 treated with DNP and CDDP—the ROS level was significantly upregulated by DNP (Figure 5C,D, *p* < 0.001). Elevated intracellular ROS is also a hallmark of early apoptosis [23], which may be one of the reasons why DNP promotes more apoptosis. Indeed, DNP exhibits a synergistic effect with CDDP and NPX, which allows for the accumulation of ROS above the threshold for monotherapy. This, in turn, enhances oxidative stress-mediated toxicity. To evaluate the anti-inflammatory potential of DNP, we established an lipopolysaccharide (LPS)-induced macrophage activation model using RAW264.7 cells. Quantitative analysis demonstrated significant suppression of the mRNA expression of pro-inflammatory cytokine (IL-6, IL-1β, TNF-α) in DNP-treated group compared to CDDP-treated group (Figure 5E). This finding indicates that DNP offers supplementary COX inhibitors, which markedly suppress the generation of numerous inflammatory mediators.

### 2.6. The In Vivo Anti-Tumor Effected of DNP

**Figure 6:**
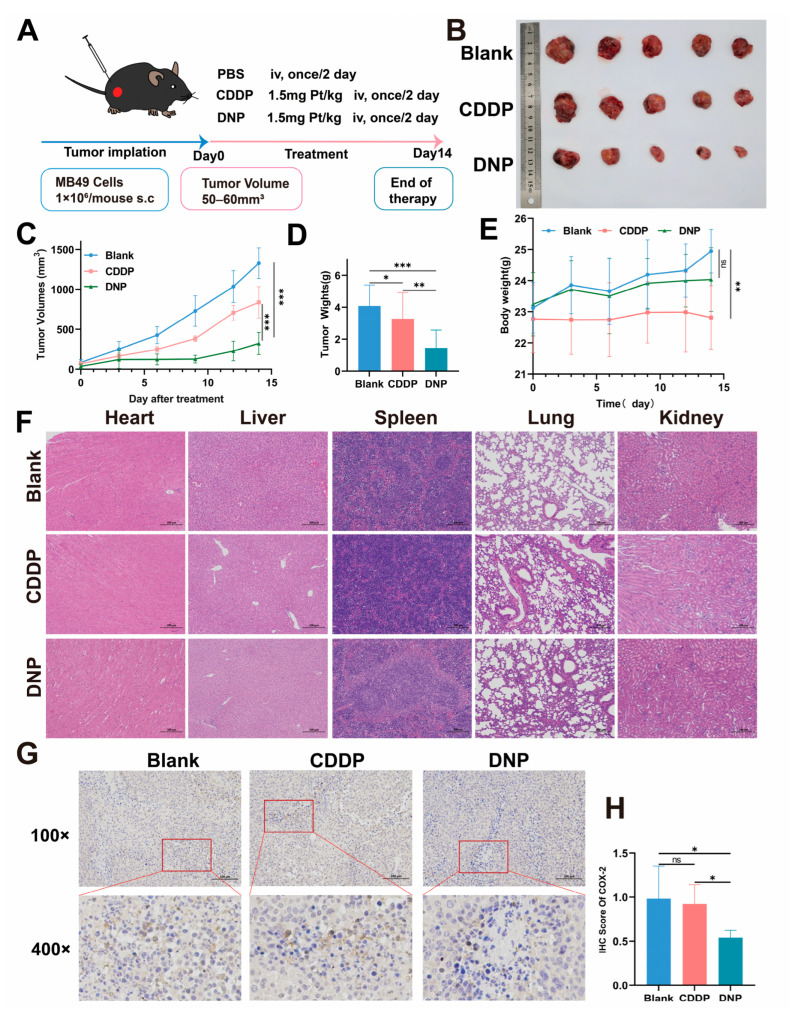
In vivo anti-tumor activity of DNP. (**A**) Experiment scheme of the MB49 tumor-bearing C57BL/6 male mouse model. (**B**) Photographs of isolated tumors after 14 days treatment (*n* = 5). (**C**) Tumor volume in mice during treatment. (**D**) Actual weight of tumors after treatment (*n* = 5). (**E**) Weight changes in mice during treatment (*n* = 5). (**F**) H&E staining of heart, liver, spleen, lungs, and kidneys in tumor-bearing mice post-treatment. (**G**) Representative images of COX-2 immunohistochemistry in mouse tumor tissues. (**H**) Quantitative analysis of COX-2 expression levels. ns *p* > 0.05; * *p* < 0.05; ** *p* < 0.01; *** *p* < 0.001.

The antitumor efficacy and safety profile of DNP were systematically evaluated in an orthotopic MB49 bladder cancer model over a 14-day therapeutic course (Figure 6A). After the monitoring of tumor growth, DNP (Pt-equivalent dose to cisplatin) demonstrated superior suppression compared to cisplatin (Figure 6B,D, *p* < 0.01). Notably, DNP-treated mice maintained stable body weights throughout the study (*p* = 0.484), contrasting with cisplatin-induced weight loss (Figure 6E, *p* < 0.01). Histopathological assessment of major organs demonstrated DNP’s favorable safety profile: H&E staining showed preserved tissue architecture in heart, liver, spleen, and lungs, with no evidence of cisplatin-associated nephrotoxicity in DNP group (Figure 6F). Mechanistically, immunohistochemical analysis revealed significant downregulation of COX-2 expression in DNP-treated tumors, correlating with its antitumor activity (Figure 6G, *p* < 0.05). This dual functionality—potent COX-2 pathway inhibition coupled with platinum-mediated cytotoxicity—positions DNP as a promising therapeutic candidate with enhanced efficacy–toxicity ratio for bladder cancer management.

## 3. Discussion

Bladder cancer is a high-grade urological tumor of high malignancy with high mortality and recurrence rates [24]. Over the past three decades, bladder cancer clinical management has evolved from radical cystectomy to platinum-based neoadjuvant chemotherapy combined with radiotherapy, representing a paradigm shift in treatment strategies. However, key prognostic indicators such as the five-year survival rate have not improved significantly [25,26]. Previous studies have reported that traditional divalent platinum-based chemotherapeutic agents (e.g., cisplatin, carboplatin, oxaliplatin) often induce severe side effects and drug resistance [27,28,29]. With advances in nanotechnology and chemical synthesis, novel platinum-based drugs and their targeted modifications undoubtedly offer promising prospects for improving current anticancer therapies [30]. Platinum(IV) complexes, serving as prodrugs for divalent platinum agents, may address this clinical challenge through rational targeted modifications.

We demonstrated that DNP exhibited significantly stronger cytotoxicity than cisplatin. Previous studies have reported that DNP exhibits significantly higher lipophilicity (logPO/W = 1.46) than cisplatin (logPO/W = −2.35) [18]. Given that the lipophilicity of compounds is directly correlated with their passive diffusion efficiency across membranes, the enhanced hydrophobicity of DNP facilitates its penetration through the cellular membrane barrier, leading to a significant increase in intracellular platinum accumulation. This observation aligns well with our subsequent findings: compared to cisplatin, DNP demonstrates significantly higher platinum uptake in cancer cells and greater platinum content bound to cellular DNA. Furthermore, DNP exhibited significant growth inhibition not only in monolayer-cultured cancer cells but also in three-dimensional (3D) tumor spheroids. This benefit can undoubtedly be attributed to the enhanced lipophilicity of DNP, which facilitates its penetration through the outer layers of tumor spheroids. Moreover, colony formation assays revealed that cancer cells treated with DNP exhibited nearly complete growth inhibition.

Moreover, we used RNA-seq technology to study T24 cells treated with DNP and cisplatin and found that DNP has a unique multi-target mechanism. Both KEGG and GSEA analyses consistently showed that DNP triggered pathways related to ROS (like oxidative phosphorylation and apoptosis) and also blocked TNF signaling, which is an inflammatory pathway. This finding provides direct evidence supporting the “chemo-anti-inflammatory” dual-functional design concept for Pt(IV) prodrugs. Subsequent apoptosis assays revealed that DNP exhibited significantly higher pro-apoptotic efficiency than cisplatin. This improvement might come from DNP’s special redox properties, which greatly raise ROS levels and directly activate the mitochondrial apoptosis pathway, as noted in earlier studies [23]. Furthermore, DNP demonstrated significant anti-inflammatory activity in the inflammatory model, which may be due to its axial ligand NPX blocking prostaglandin synthesis. However, the study has the following limitations: the quantitative relationship between ROS levels and apoptosis rates needs to be validated, and the precise mechanism of the axial ligand NPX needs to be clarified. In summary, DNP exerts its antitumor effects through a unique dual mechanism involving both pro-apoptotic and anti-inflammatory actions. This distinctive property suggests its potential to overcome the drug resistance and toxicity limitations associated with conventional platinum-based agents.

Finally, we established an orthotopic bladder cancer animal model to evaluate the therapeutic potential of the novel platinum(IV) complex DNP. The findings provide important guidance for the clinical translation of platinum-based drugs. DNP demonstrated significantly superior antitumor efficacy compared to cisplatin, which may be attributed to its unique mechanism of action. The enhanced lipophilicity of the Pt(IV) prodrug likely improves tumor tissue penetration and retention effects. Additionally, the axial NPX ligand may potentiate the platinum core’s cytotoxicity by downregulating COX-2 expression. Histopathological examination of organ tissues demonstrated superior safety profiles of DNP, as evidenced by the absence of cisplatin-typical renal tubular vacuolization [31] and well-preserved histological integrity in major organs. Interestingly, no significant changes in body weight were observed in model mice. Our study provides critical evidence for the further development of DNP, but future investigations are still required to characterize its pharmacokinetic profile and safety in vivo.

## 4. Materials and Methods

### 4.1. Reagents and Chemicals

Cisplatin was purchased from MCE. Hydrogen peroxide (30%, H_2_O_2_), nitric acid, and hydrochloric acid were purchased from Sinopharm Chemical Reagent. The remaining chemical reagents came from Aladdin Biochemical Technology Co., Ltd. (Shanghai, China), while dimethyl sulfoxide (DMSO) was purchased from Sigma (St. Louis, MO, USA).

RPMI 1640 medium, Dulbecco’s modified Eagle’s medium (DMEM) (high glucose), fetal bovine serum (FBS) and penicillin-Streptomycin were purchased from Gibco (Oakland, CA, USA) Trypsin without EDTA (FMS20231201001) was purchased from Fcmacs Biotech Co., Ltd (Nanjing, China). The Cell Counting Kit-8 (CCK-8, KTA1020-1000T) was purchased from Abbkine Scientific Co., Ltd (Wuhan, China). The Calcein/PI Cell Viability and Cytotoxicity Assay Kit (C2015S), the BCA Protein Assay Kit (P0010S), and the Reactive Oxygen Species Detection Kit (S0033S) were purchased from Beyotime Biotechnology (Shanghai, China). The genomic DNA extraction kit (DC102-01) for cells and tissues, CellCounting-Lite 3D Luminescent Assay (Vazyme #DD1102) as well as the qPCR reagents, were purchased from Vazyme Biotech Co., Ltd (Nanjing, China). Additionally, the Annexin V-FITC/PI apoptosis detection reagent (K2003) was purchased from APExBIO Technology LLC (Houston, TX, USA).

### 4.2. Cell Culture

The human bladder cancers (T24, UMUC3, J82) were obtained from the Institute of Biochemistry and Cell Biology (Shanghai, China). The murine bladder cancer cell line MB49 was generously gifted by Professor Haibo Shen (Shanghai Jiao Tong University, Shanghai, China). RAW264.7 cells were obtained from Nanjing University School of Life Sciences. The MB49 were cultured in RPMI-1640 supplemented with 10% FBS and 1× penicillin-Streptomycin, whereas the other cell lines were cultured in DMEM also enriched with 10% FBS. Cells were grown at 37 °C in an incubator (Thermo Fisher Scientific, Waltham, MA, USA) containing 5% CO_2_.

### 4.3. Animals

Six- to eight-week old male C57BL/6 mice (18–23 g) were purchased from GemPharmatech Co., Ltd (Nanjing, China). All animal experimental protocols followed the regulations of the People’s Republic of China on the Administration of Laboratory Animals, and it use protocol was reviewed and approved by the Nanjing University Animal Ethical and Welfare Committee (Approval No: IACUC-2412011). All animals were housed under a pathogen-free condition in groups of five mice per cage. The temperature was kept at 21 ± 2 °C, relative humidity varied between 40% and 70%, and a 12 h light/dark cycle was maintained.

### 4.4. Compound Synthesis·and·Characterization

The synthetic route and characterization data for compound DNP are available in the literature procedure [18]. Oxoplatin was stirred with NPX, TEA, and TBTU in DMF under dark conditions for 48 h. The resulting mixture was treated with ethanol and water to yield a yellow precipitate, which was subsequently dissolved in methanol and precipitated with diethyl ether. After several repetitions of this precipitation procedure, the product was dried under vacuum to afford a pale yellow powder.

The structural integrity and purity of the synthesized complexes were systematically characterized using an integrated analytical approach. Nuclear magnetic resonance (NMR) spectra (^1^H, ^13^C, and ^195^Pt) were acquired on a Bruker DRX-400 spectrometer (BRKR, Billerica, USA) at 298 K, with DMSO-d_6_ as the solvent and tetramethylsilane (TMS) as an internal reference. High-resolution Mass Spectrometry (HR-MS) data were measured in negative ion mode using a Thermo Q Exactive Orbitrap (Thermo Fisher Scientific, Waltham, MA, USA) instrument. Isotopic distribution patterns were simulated using ISOPRO 3.0 to confirm molecular composition. Compound purity was analyzed on a Thermo Scientific™ (Thermo Fisher Scientific, Waltham, MA, USA) Vanquish Core system equipped with a Thermo Scientific Acclaim120 C18 column (5 μm, 4.6 × 250 mm). The mobile phase comprised water (A) and acetonitrile (B) at 1.0 mL/min. Gradient elution: 0–2 min: 5% B, 2–30 min: 5–95% B, 30–35 min: 95% B. Detection at 254 nm with column temperature maintained at 40 °C. DNP was dissolved in acetonitrile (with trace DMSO for solubility), filtered through a 0.22 μm membrane, and injected (10 μL).

### 4.5. Cell Uptake

Four bladder tumor cell lines (T24, UMUC3, J82, and MB49) were seeded into 6-well plates and incubated overnight. The day after, cells were treated with DNP or cisplatin (CDDP) 12 h, with PBS-treated cells as controls. Post-treatment, cells were washed twice with ice-cold PBS, pelleted by centrifugation, and digested sequentially with nitric acid (95 °C, 2 h), hydrogen peroxide (1 h), and hydrochloric acid (1 h). Platinum content in the digested lysates was quantified via ICP-MS after filtration.

### 4.6. DNA Platination

Following the same cell culture and treatment protocol as above, genomic DNA was extracted using the cell/tissue genomic DNA extraction kit (Vazyme Biotech Co., Ltd., Nanjing, China). DNA concentration was quantified via NanoDrop (Thermo NanoDrop 1000) spectrophotometry (260 nm). DNA samples underwent sequential digestion with nitric acid (95 °C, 2 h), hydrogen peroxide (1 h), and hydrochloric acid (1 h). DNA-bound platinum content was analyzed by ICP-MS post-filtration.

### 4.7. Cytotoxicity of DNP

Cell viability was tested using the T24, UMUC3, J82, and MB49 cell lines. CDDP was dissolved in sterile PBS, DNP was dissolved in DMSO, and the stock solutions were then diluted to the desired concentration gradient using culture medium, ensuring that the DMSO was limited to less than 0.1%. Tumor cells were seeded at the density of 3000 cells/well in 96-well plates. Cells were treated with graded concentrations of CDDP or DNP for 72 h after wall attachment. The drug-containing medium was then replaced with a mixed solution of medium and CCK8 (9:1) and incubated for 1 h. Finally, the optical density (OD) was measured at 450 nm using a Synergy multifunction microplate reader. IC_50_ values were calculated using GraphPad Prism software (version 9.40) with six parallel wells for each sample. The half-maximal inhibitory concentration (IC_50_) was calculated as follows:

$${\mathrm{IC}}_{50}\;=\;(\frac{OD\;of\;treatment\;group-OD\;of\;blank\;control\;group}{OD\;of\;control\;group-OD\;of\;blank\;control\;group})\;\times \;100\%$$

### 4.8. Live/Dead Cell Staining Method

The Calcein AM/PI cell viability detection kit was used to determine the proliferative activity of T24 cells. Tumor cells were seeded at a density of 3000 cells/well in a 96-well plate. When the cell density reached approximately 70%, they were treated with CDDP (0.2 µM), DNP (0.2 µM) for 24 h. Then, we incubated the cells with calcein-AM/propidium iodide for 30 min and imaged using a fluorescence microscope (LeicaWetzlar, Germany).

### 4.9. Colony Formation Assay

T24 cells were seeded in 6-well plates at a density of 1000 cells/well and allowed to adhere for 24 h. Cells were then exposed to CDDP (0.2 µM) or DNP (0.2 µM) for a duration of 24 h, followed by replacement with fresh DMEM. Medium was refreshed every 3 days until colonies had formed (7–10 days). Subsequent to this, the medium was discarded, and the cells were washed twice with PBS. The cells were then fixed with 4% paraformaldehyde for 15 min, after which they were stained with 0.5% crystal violet and photographed for subsequent analysis.

### 4.10. Three-Dimensional Tumor Spheroid Viability Assay

T24 cells were seeded at a density of 1000 cells/well in ultra-low-attachment 96-well plate (Corning, Glendale, AZ, USA). After the cells form stable spheroids, they are intervened with CDDP (5 µM) and DNP (5 µM) for 5 days. Then, the cell spheroids were washed 2–3 times with PBS and stained with Calcein AM/PI detection reagent. Finally, images were taken using a fluorescence microscope (LeicaWetzlar, Germany).

### 4.11. Bulk Sequencing

T24 cells were seeded at a density of 3 × 10^5^ in 6 cm culture dishes. When the cell density reaches 80%, the cells are treated with DNP (2 µM) and CDDP (2 µM) for 24 h. Later, the cell samples were collected, and total RNA was extracted using Trizol reagent according to the instructions. The RNA samples were then quantified using a NanoDrop 1000 (Thermo Fisher Scientific, Waltham, MA, USA) to ensure that the samples selected for library construction were of adequate quality. Lastly, the transcriptome sequencing analysis was performed by Singleron Biotech Co., Ltd Nanjing, China. Genes with a false discovery rate (FDR) parameter less than 0.05 and an absolute multiple change of at least 2 are considered to be differentially expressed genes (DEGs). The identification of DEGs between the CDDP group and the DNP group was facilitated by the utilization of volcano plots and heat maps. Subsequent to this, the identification of DEGs was subjected to Kyoto Encyclopedia of Genes and Genomes (KEGG) pathway enrichment analysis and gene set enrichment analysis (GSEA).

### 4.12. Cell Apoptosis Assay

T24 cells were seeded at a density of 2 × 10^5^ cells/well in 6-well plates. When the cell density reached 70%, the cells were incubated with DNP (0.1 µM) and CDDP (0.1 µM) for 48 h. The cells were then collected using trypsin without EDTA (Fcmacs) and washed twice with ice-cold PBS. The apoptosis rate was detected using the Annexin V-FITC/PI Apoptosis Kit (APExBIO Technology LLC (Houston, TX, USA). Following the kit instructions to add Annexin V-FITC and PI, cells were incubated in the dark for 15 min. After washing twice with PBS, the resuspended cells were prepared for flow cytometry analysis (Beckman Coulter, Inc, Brea, CA, USA).

### 4.13. RT-qPCR Analysis

RAW264.7 cells were seeded at a density of 5 × 10^4^ cells/well in 12-well plates and stimulated with lipopolysaccharide (LPS, 100 ng/mL) for 18 h. Then, RAW264.7 cells were treated with CDDP (0.4 µM) and DNP (0.4 µM) for 18 h. The treated cells were collected, and whole RNA was extracted and detected according to the above method. Subsequently, HiScript II Q RT SuperMix for qRT-PCR (+gDNA wiper) (Vazyme Biotech Co., Ltd, Nanjing, China) was utilized for reverse transcription, and ChamQ Blue Universal SYBR qPCR Master Mix (Vazyme, Q312) for PCR amplification. The reaction conditions were as follows: Initial denaturation at 95 °C for 30 s; then 95 °C for 10 s, 60 °C for 30 s for a total of 40 cycles; then 95 °C for 15 s, 60 °C for 1 min; finally 95 °C for 15 s. Utilizing GAPDH as the reference gene, the mRNA expression levels of the pro-inflammatory cytokines TNF-α, IL-1β, and IL-6 in the cells were measured employing the 2-ΔΔCT method. 

### 4.14. ROS Production Assay

T24 cells were seeded at a density of 2 × 10^5^ cells/well in 6-well plates. When the cell density reaches 70%, the cells are incubated with DNP (0.2 µM) and CDDP (0.2 µM) for 24 h. Tumor cells were collected and washed twice with PBS. The level of ROS produced in T24 cells was detected using a reactive oxygen detection kit. The cells were incubated with 10 µM DCF-DA probe in the dark for 30 min, washed 2–3 times with FBS-free medium, and resuspended for flow cytometry analysis.

### 4.15. Establishment and Treatment of Subcutaneous Tumor Model

The design of the in vivo treatment experiment is shown in Figure 6A. 1 × 10^6^ MB49 cells were injected into the right flank. The tumor volume and body weight were evaluated every two days. Until the tumors grew to approximately 50 mm^3^, mice were randomized, and the first treatment was administered. All animals were divided into three groups (*n* = 5) and treated with PBS (control group), CDDP (1.5 mg Pt/kg), or DNP (1.5 mg Pt/kg). The drug was administered intravenously every two days until the tumor volume of the control group reached 1500 mm^3^. After the treatment, all mice were executed and the tumor tissue as well as vital organs (heart, liver, spleen, lungs, and kidneys) was surgically excised.

### 4.16. Hematoxylin and Eosin Staining

The microtome (Leica, Wetzlar, Germany) was utilized to achieve 5-micron-thick sections from the paraffin-embedded mouse tumor tissues. The tissue sections should then be placed in an oven (GNP-9050, Shanghai, China) at 65 °C for 1 h. The deparaffinization of the sections was then conducted using xylene, followed by hydration with a series of ethanol solutions, ranging from 100% to 30%. The next step is to stain the sections with hematoxylin solution for 5 min, differentiate them in 0.25% hydrochloric acid in ethanol for 5 s, and then rinse them in distilled water. Following this, the sections should be stained with eosin solution for 5 s and then rehydrated through a series of progressively higher alcohols (from 75% to 100%) and xylene. Finally, the slides should be photographed using a fluorescence microscope (Leica, Wetzlar, Germany).

### 4.17. Immunohistochemistry (IHC)

Firstly, the mouse tumor tissues must be deposited in an oven that has been calibrated to 65 °C for a period of 20 min. Subsequently, the sections should be deparaffinised using xylene and hydrated through a graded series of alcohol (from 100% to 70%), followed by rinsing with distilled water. The next step is to place the slides in an antigen retrieval solution at 95 °C and incubate for 10 min to complete antigen retrieval. The slides were then left at room temperature for ten minutes to soak in 3% hydrogen peroxide, which inhibited the activity of endogenous peroxidase. The slides were then washed thrice with PBS and subsequently blocked with a 5% bovine serum albumin (BSA) solution at ambient temperature for a period of one hour. The main antibody against COX-2 was then incubated with the slides for one hour at 37 °C in a humidified box. This was followed by three washes with PBST. Then, an appropriate dilution of secondary antibody was incubated with the slides for 30 min at 37 °C in a humidified box. Staining was then performed with 3,3′-diaminobenzidine (DAB) and hematoxylin. Finally, the samples should be photographed using a microscope (Leica, Wetzlar, Germany).

### 4.18. Statistical Analysis

Data analysis was conducted with GraphPad Prism 8.0, with results presented as mean ± standard deviation. Intergroup comparisons utilized one-way ANOVA, with statistical significance thresholds defined as * *p* < 0.05, ** *p* < 0.01, and *** *p* < 0.001, **** *p* < 0.0001.

## 5. Conclusions

This study demonstrates that the Pt(IV) prodrug DNP could represent a paradigm shift in bladder cancer therapy by synergistically integrating platinum-mediated cytotoxicity with naproxen driven anti-inflammatory action. Unlike conventional cisplatin, DNP leverages tumor microenvironment-responsive activation to selectively release platinum species and NPX within malignant tissues, which not only enhances therapeutic efficacy but also circumvents systemic toxicity. The intrinsic anti-inflammatory properties of DNP disrupt COX-2-mediated inflammatory cascades, which are known to promote tumor progression and chemoresistance in bladder cancer. Furthermore, the coordinated release of bioactive components creates a self-amplifying therapeutic cycle, where platinum-induced oxidative stress potentiates anti-inflammatory effects, while the production of ROS sensitizes cells to apoptosis. Importantly, DNP’s tumor-selective activation preserves normal tissue integrity, addressing the nephrotoxicity that plagues cisplatin-based regimens. While this prodrug strategy shows transformative potential, future work should explore its applicability in chemoresistant models and compatibility with emerging immunotherapies. In summary, these data demonstrate that DNP exhibits superior antitumor activity in cancer cells, providing compelling experimental evidence for subsequent in vivo studies.

## Figures and Tables

**Table 1:** In Vitro Cytotoxicity: IC_50_ Values of DNP, CDDP, and CDDP/NPX mixture in Bladder Cancer Cell Lines (72 h).

Cell Lines	IC_50_ of Complexes (µM)			
	DNP	CDDP	CDDP + NPX	NPX
T24	0.11 ± 0.004	3.75 ± 0.02	5.29 ± 0.07	>100
UMUC3	0.18 ± 0.02	4.63 ± 0.21	5.57 ± 0.77	>150
J82	0.18 ± 0.009	1.90 ± 0.02	2.64 ± 0.14	>120
MB49	0.54 ± 0.07	6.36 ± 0.44	5.22 ± 0.26	>150

## Data Availability

The original data are available from the corresponding authors upon reasonable request.

## Supplementary Materials

The following supporting information can be downloaded at: https://www.mdpi.com/article/10.3390/ph18081185/s1, Scheme S1: Synthetic routes to DNP; Figure S1: ^1^H NMR of DNP (400 MHz, DMSO-d_6_); Figure S2: ^13^C NMR of DNP (101 MHz, DMSO-d_6_); Figure S3: ^195^Pt NMR spectrum of DNP (86 MHz, DMSO-d_6_); Figure S4: HR-MS (negative mode) spectra of DNP; Figure S5: HPLC chromatograms of DNP.

## Abbreviations

Pt(IV)	Platinum(IV)
COX-2	Cyclooxygenase-2
LPS	Lipopolysaccharide
CDDP	Cisplatin
NSAIDs	Nonsteroidal anti-inflammatory drugs
NPX	Naproxen
DMSO	Dimethylsulfoxide
PBS	Phosphate-buffered saline
TNF-α	Tumor necrosis factor-α
IL-1β	Interleukin-1β
IL-6	Interleukin-6
ROS	Reactive oxygen species
IHC	Immunohistochemical
H&E	Hematoxylin–eosin
ANOVA	Analysis of variance
