# Supplementary material for: A COX-2-Targeted Platinum(lV) Prodrug Induces Apoptosis and Reduces Inflammation in Bladder Cancer Models
Source: Pharmaceuticals (Basel). 2025 Aug 12;18(8):1185. doi: 10.3390/ph18081185 (PMC12388935; doi:10.3390/ph18081185)
Supplement: Supplementary file 1 [file pharmaceuticals-18-01185-s001.zip › pharmaceuticals-3774532-supplementary.pdf]

Electronic Supporting Information

for

Novel Multitargeted Platinum(IV) Complex Attenuates Bladder Cancer Progression Through Apoptotic Induction And Inflammatory Intervention

Experimental Details

Scheme S1. Synthetic routes to DNP

Figure S1. ^{1}H NMR of Pt- naproxen (DNP)

Figure S2. ^{13}C NMR of DNP

Figure S3. ^{195}Pt NMR of DNP

Figure S4. HR-MS (negative mode) spectra of DNP

Figure S5. HPLC chromatograms of DNP

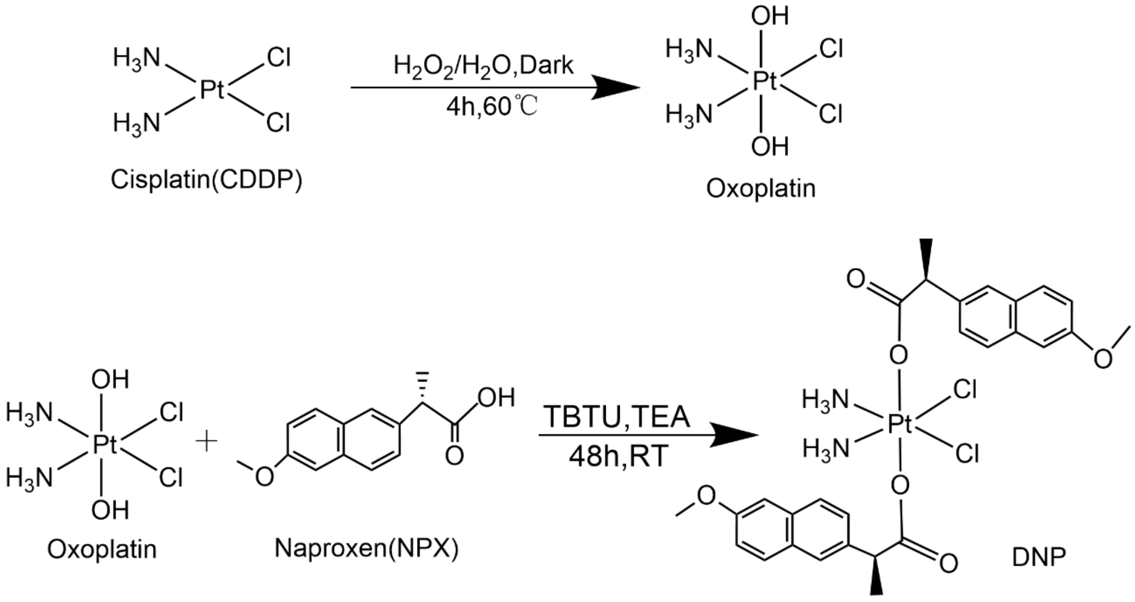

Scheme S1. Synthetic routes to DNP.

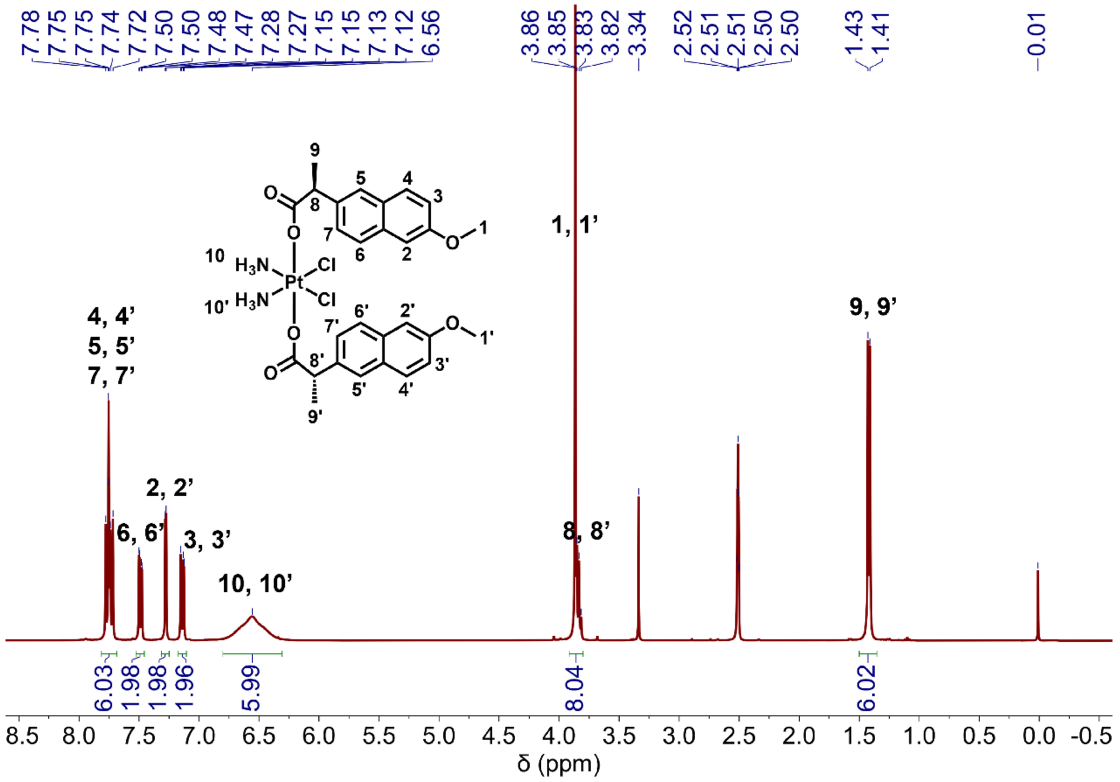

Figure S1. ^{1}H NMR of DNP (400 MHz, DMSO-d_6).

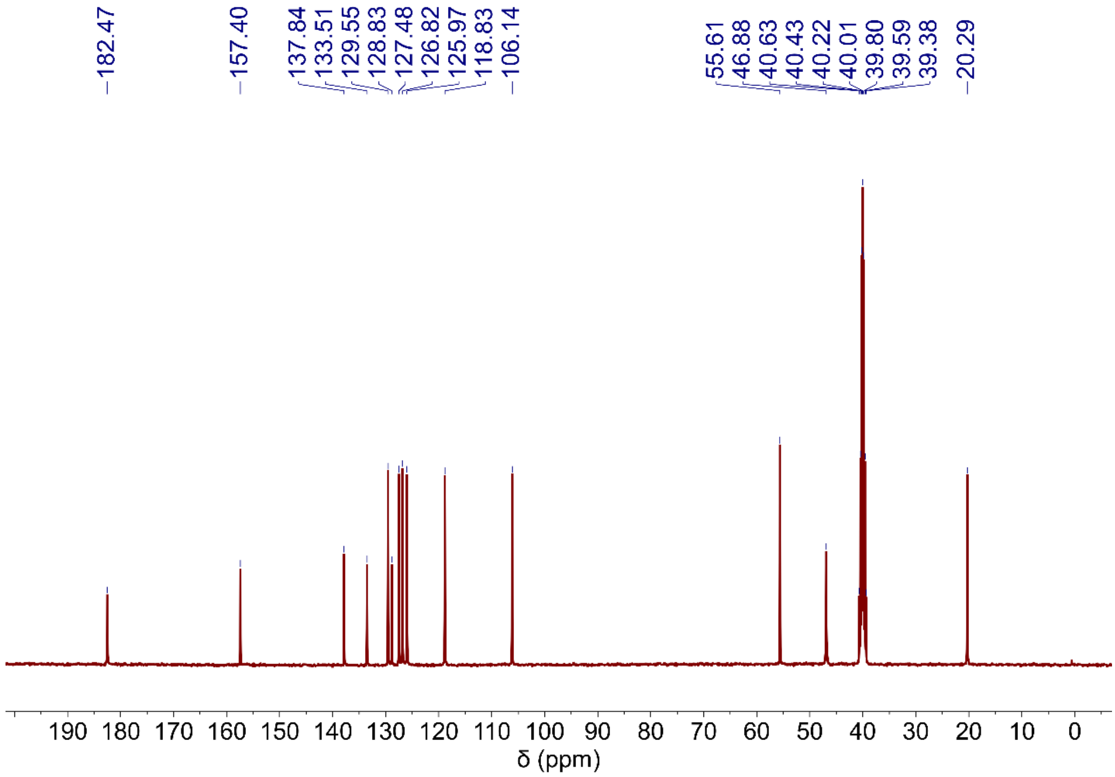

Figure S2. ^{13}C NMR of DNP (101 MHz, DMSO-d_6).

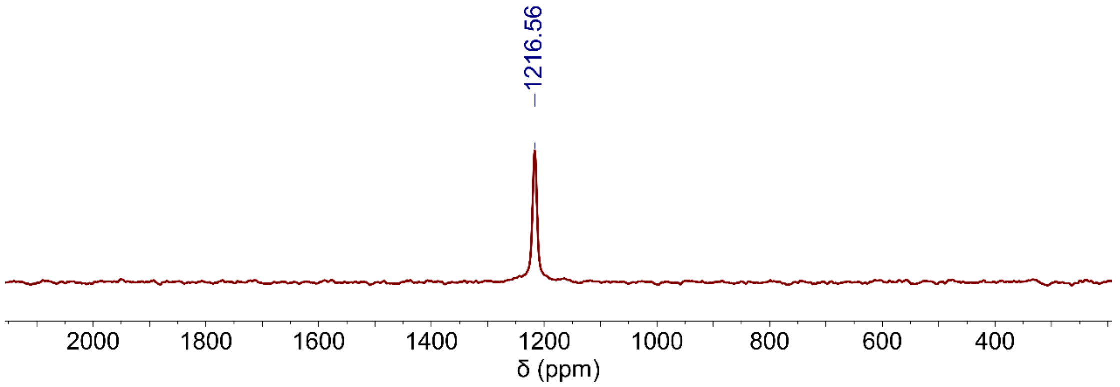

Figure S3. ^{195}Pt NMR spectrum of DNP (86 MHz, DMSO-d_6).

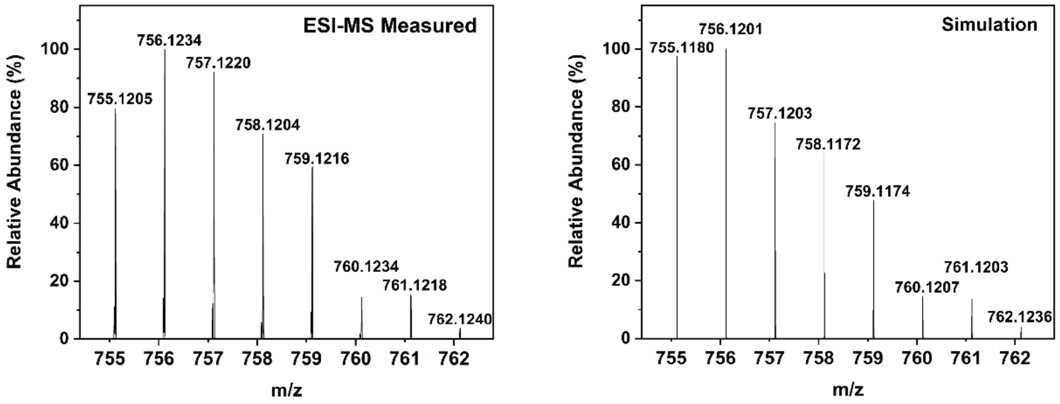

Figure S4. HR-MS (negative mode) spectra of DNP.

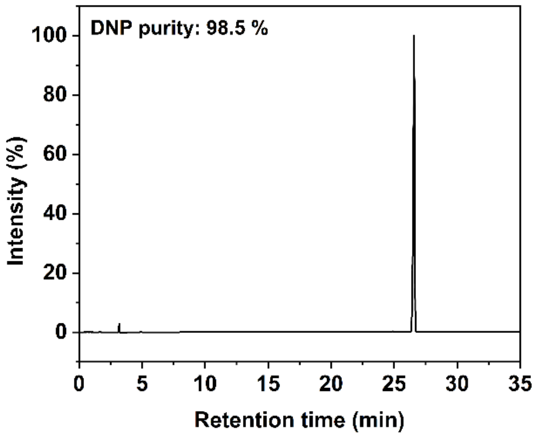

Figure S5. HPLC chromatograms of DNP.
